# A Selective Role of Dietary Anthocyanins and Flavan-3-ols in Reducing the Risk of Type 2 Diabetes Mellitus: A Review of Recent Evidence

**DOI:** 10.3390/nu11040841

**Published:** 2019-04-13

**Authors:** Britt Burton-Freeman, Michał Brzeziński, Eunyoung Park, Amandeep Sandhu, Di Xiao, Indika Edirisinghe

**Affiliations:** 1 Department of Food Science and Nutrition, Center for Nutrition Research, Institute for Food Safety and Health, Illinois Institute of Technology, Chicago, IL 60616, USA; brzezinski@gumed.edu.pl (M.B.); epark4@iit.edu (E.P.); asandhu2@iit.edu (A.S.); dxiao6@iit.edu (D.X.); iedirisi@iit.edu (I.E.); 2 Department of Public Health and Social Medicine, Medical University of Gdansk, 80-210 Gdansk, Poland

**Keywords:** flavonoids, polyphenols, insulin, glucose, diabetes, glycemic control

## Abstract

Type 2 diabetes mellitus (T2DM) is the most common form of DM and its prevalence is increasing worldwide. Because it is a progressive disease, prevention, early detection and disease course modification are possible. Diet plays a critical role in reducing T2DM risk. Therapeutic dietary approaches routinely recommend diets high in plant foods (i.e., vegetables, fruits, whole-grains). In addition to essential micronutrients and fiber, plant-based diets contain a wide-variety of polyphenols, specifically flavonoid compounds. Evidence suggests that flavonoids may confer specific benefits for T2DM risk reduction through pathways influencing glucose absorption and insulin sensitivity and/or secretion. The present review assesses the relationship between dietary flavonoids and diabetes risk reduction reviewing current epidemiology and clinical research. Collectively, the research indicates that certain flavonoids, explicitly anthocyanins and flavan-3-ols and foods rich in these compounds, may have an important role in dietary algorithms aimed to address diabetes risk factors and the development of T2DM.

## 1. Introduction

Diabetes Mellitus (DM) is a complex metabolic disease characterized by hyperglycemia resulting from impairments in insulin secretion, insulin action, or both [1]. The number of individuals with DM has increased nearly four-fold since 1980 from 108 million to ~422 million worldwide [2]. The health care liability of DM in the United States of America (USA) and other parts of the world has increased accordingly [3,4]. People with DM have an increased risk of developing a number of health issues. Persistently elevated blood glucose causes generalized vascular damage affecting the heart and blood vessels, eyes, kidney and nerves [5]. While Type 1 DM cannot be prevented with existing knowledge, prevention is possible for Type 2 DM (T2DM), and management of any DM reduces risk and severity of complications and premature death. Identifying early indicators of metabolic disruption and intervening with effective affordable approaches is critical in preventing T2DM and reducing the associated individual, national and global health care burden. 

## 2. Path to T2DM

**Figure 1:**
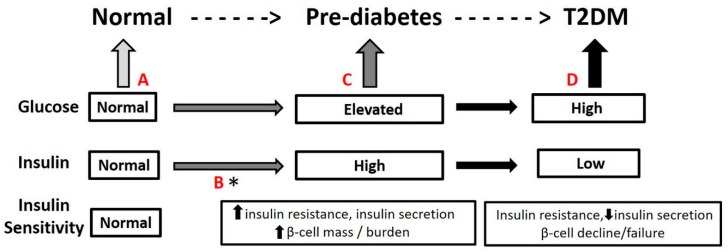
Path to Diabetes/T2DM: **A**. normal, non-disease/diabetes state (based on fasting glucose 70–99 mg/dL). **B**. declining insulin sensitivity/increasing insulin resistance, and increasing concentrations of insulin (hyperinsulinemia) to maintain normal glucose: Opportunity * for early detection and intervention to restore normal insulin sensitivity and decrease beta cell burden. **C**. pre-diabetes identified (fasting glucose 100–125 mg/dL, [1], hyperinsulinemia insufficient to overcome insulin resistance. Diet and lifestyle intervention prescribed. **D**. T2DM diagnosed (fasting glucose ≥126 mg/dL or 2-h post glucose ≥140 mg/dL, [1], diet and lifestyle and medications prescribed.

T2DM is the most common form of DM. Although both genetic and environmental factors contribute to the development and progression of T2DM [6], growing prevalence seems to be the result of changing dietary habits and lifestyles observed in modern societies [7]. Many of these changes promote obesity development, an established risk factor for T2DM [7,8]. Increasing body mass index (BMI) is associated with central adiposity, low-grade chronic inflammation, and cellular redox imbalances that lead to impaired metabolic processing. Impairments in metabolism are largely the result of tissue resistance to insulin’s actions [9,10], and may be considered the first stage along the disease path. This first stage is characterized by a long period of insulin resistance and compensatory hyperinsulinemia to control blood glucose within normal ranges (Figure 1A→B). In the progressive path to T2DM, insulin resistance continues to increase until insulin secretion from the pancreatic β-cell fails to compensate fully resulting in elevated blood glucose concentrations that eventually meet criteria for pre-diabetes (100–125 mg/dL, Figure 1C, [1]). Pre-diabetes represents a critical crossroads as ~50% of those with pre-diabetes will develop T2DM within seven years and 83% will convert over a lifetime (Figure 1D) [11]. Although the path to T2DM begins early with insulin resistance (Figure 1A→B), preventative care does not usually start until pre-diabetes is identified (Figure 1C). Intervention strategies typically focus on diet and lifestyle modification [12,13,14,15], yet, arguably, these strategies could begin sooner when metabolic impairments first appear, such as with insulin resistance (Figure 1B) [9]. Identifying specific dietary flavonoids that act on these early indicators is opportunistic and would deliver targeted dietary modification for overall disease risk reduction. 

## 3. Diet and T2DM

The Western/modern day diet has been a major factor in the increased incidence of T2DM [12]. In addition to excess calorie intake, food choices are nutrient poor and comprised of highly refined, readily available carbohydrates and fats [14]. Modification of diet and lifestyle has proven to be a principal approach in T2DM risk reduction: the clinical evidence demonstrating these modifications were more effective than medication (metformin) in preventing conversion from pre-diabetes to T2DM in the Diabetes Prevention Program clinical trial (*n* = 3234) [16]. Dietary patterns, such as the Mediterranean diet and the Dietary Approaches to Stop Hypertension (DASH) diet also have shown favorable outcomes in T2DM risk reduction [17,18]. The foundation of these diets is plant foods (i.e., vegetables, fruits and whole-grains), which contain varied essential nutrients and fiber [19,20,21], along with an array of phytochemicals with biological activity. Some of these bioactive compounds are suggested to have glucose modulating and insulin sensitizing effects at the cellular level. Polyphenols, and specifically flavonoid compounds, are among the most studied for their biological activity and some may have an important role in preventing or delaying the onset of T2DM.

## 4. Dietary Flavonoids and T2DM 

### 4.1. Flavonoids: General Chemistry and Intake

**Figure 2:**
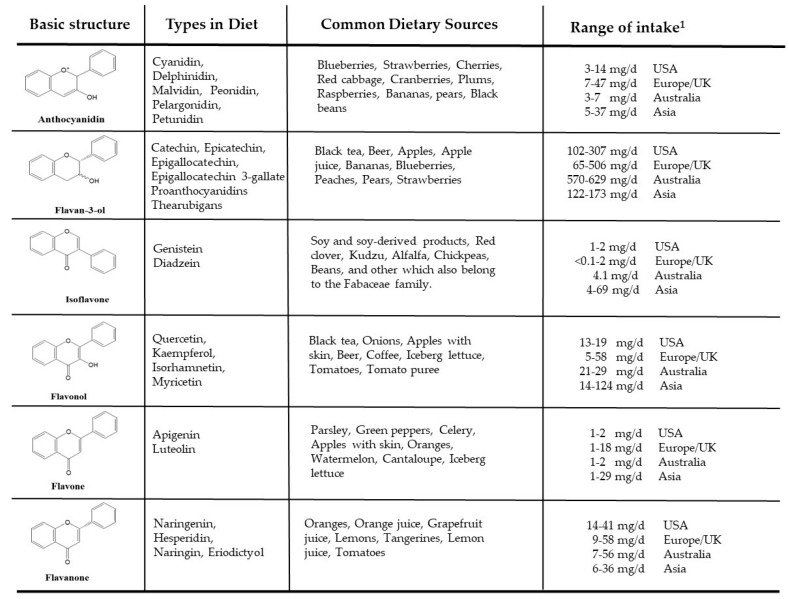
Flavonoid compounds, dietary sources and daily intake range. ^1^ Aglycone values [25].

Flavonoid compounds are widely distributed in plant foods and represent ~2/3 of the total dietary polyphenols consumed [22]. Flavonoids are secondary plant metabolites with a characteristic C6–C3–C6 structural backbone. They are classified into six main categories: flavone, isoflavone, flavan-3-ol, flavanone, anthocyanidin and flavonol. More than 9000 flavonoids have been reported [23] differing in degree of hydroxylation and methylation of A and B rings along with the presence of various glycosylation patterns (O- or C-glycosides). Intake of dietary flavonoids ranges from 209 to 1017 mg/d (mean 435 mg/d) in Australian, European, and USA adult populations [24]. Figure 2 provides a summary of flavonoid compounds, dietary sources, and ranges of intake [24,25,26].

### 4.2. Epidemiology of Dietary Flavonoids and T2DM Incidence

The collective epidemiological research suggests an inverse association between dietary flavonoids and T2DM incidence [27,28,29,30,31]. This association appears to be driven by a few select flavonoids [28,29,30,31]. Dietary anthocyanins and flavan-3-ols have been consistently identified as having a strong association with T2DM risk reduction [30,31], and flavonols and isoflavones have shown risk reduction associations intermittently and with less strength. These assertions are further supported by observational data linking foods relatively high in these compounds, such as berries, tea, and chocolate, and reduced incidence of T2DM [28,32,33,34,35,36,37,38,39,40,41]. Dose–response meta-analyses have suggested that 7.5 mg/day increment of dietary anthocyanin intake or 17 g/d berry intake could decrease the risk of T2DM by 5% [28] and ≥3 cups of tea/day [33] or 1–6 servings of chocolate per week were related to significant risk reduction [40].

How flavonoids, and specifically those with the strongest relationship, anthocyanins and flavan-3-ols, reduce the incidence of T2DM is not clear. Risk factor modulation is one hypothesis. Insulin resistance is a major risk factor of T2DM, and the links between excess adiposity, chronic low grade inflammation and cellular oxidative stress in insulin resistance pathogenesis are well established [42,43,44]. Flavonoids are suggested to have anti-obesity, anti-oxidant and anti-inflammatory effects. Increased consumption of anthocyanins, flavan-3-ols, flavonols, and flavonoid polymers was inversely associated with weight gain over four-year time intervals in men and women of USA [45] and, after adjustment for dietary fiber, only anthocyanins and total flavonoid polymers remained significant. In a study using data from the Twins United Kingdom (UK) registry, flavonoid subclasses including anthocyanins, flavan-3-ols, and flavonols, were associated with a more favorable fat mass distribution as measured by dual-energy X-ray absorptiometry [46]. Importantly, these effects were independent of genetic and common environmental factors, as well as after controlling for total fruit and vegetable, and fiber intake. Also using the UK Twins registry, Jennings et al. examined the impact of individual flavonoid subclasses on various indicators of insulin action and inflammation [47]. They reported an inverse relationship between dietary anthocyanins and flavones and insulin resistance, fasting insulin and markers of inflammation in women 18–76 years old [47]. In another cross-sectional analysis of 2375 Framingham Heart Study Offspring Cohort participants, higher anthocyanin and flavonol intakes were associated with significantly lower composite inflammation scores [48], with anthocyanins having markedly stronger (>2 fold) association than flavonols in the fully adjusted model. When sub-scores were analyzed further, higher anthocyanin intake was significantly and inversely associated with all sub-scores, including biomarker concentrations contributing to acute inflammation, oxidative stress and cytokine levels. Higher flavonol intake was associated with lower cytokine and oxidative stress biomarker concentrations, and flavan-3-ols and total flavonoids were inversely associated with oxidative stress markers [48]. Overall, the epidemiological findings strongly support a role of certain flavonoids in reducing the incidence of T2DM, and the effect may be achieved through reducing inflammation, oxidative stress and insulin resistance. 

### 4.3. Mechanisms of Action of Flavonoids with Anti-Diabetic Effects

**Figure 3:**
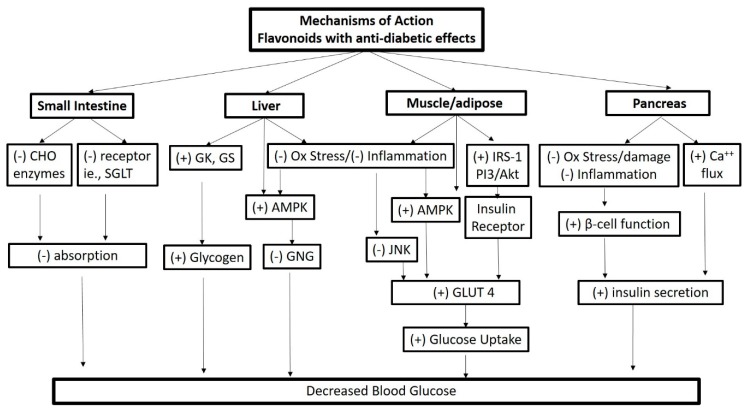
Purported mechanisms of action of flavonoids with effects on glucose metabolism: AMPK, AMP-activated protein kinase; CHO, carbohydrate; GK, Glucokinase; GLUT 4, Glucose transporter type 4; GNG, Gluconeogenesis; GS, Glutamine synthetase; IRS-1, Insulin receptor substrate 1; JNK, c-Jun N-terminal kinases; Ox, Oxidative; PI3/AKT, phosphatidylinositol 3-kinase (PI3K) and AKT/Protein Kinase B signaling pathway; SGLT, Sodium-dependent glucose transporter; (+), increase, activation; (−), decrease, inactivation.

The anti-diabetic effects of compounds, foods, and diets culminate to support glucose homeostasis through a number of possible mechanisms. Figure 3 summarizes purported mechanisms of action of flavonoids proposed to have anti-diabetic effects. In general, their bioactivity can be attributed to modulation of insulin dependent or independent pathways to decrease blood glucose. Insulin dependent pathways involve modifying cellular redox status and cell signaling paths that influence activities such as insulin synthesis and secretion (i.e., pancreatic β-cell function) and/or peripheral insulin sensitivity via Phosphoinositide 3-kinases (PI3K)/ Protein kinase B (AKT) or Peroxisome proliferator-activated receptor gamma (PPAR-γ) activation in tissues such as muscle, adipose and others [49,50,51,52]. Modulation of insulin-independent pathways includes activation of energy sensing molecules, such as AMP-activated protein kinase (AMPK) in liver, muscle and adipose [51,52]. Interference with carbohydrate digestion and glucose absorption in the small intestine has also been described via inhibition of α-amylase and/or α-glucosidase activity [53] and/or interfering with glucose transport [54]. While present knowledge has yet to identify the dominant mechanism or the relative potency of individual flavonoids, current epidemiological research suggests that anthocyanins and flavan-3-ols are leading flavonoids underpinning the flavonoid-diabetes risk reduction relationship. Other flavonoids with possible modulatory effects in diabetes pathways include flavonols and isoflavones, and many of the same mechanisms have been identified in vitro and in vivo animal studies [49,55,56].

## 5. Selecting Flavonoids That Reduce the Risk of Developing T2DM

In the general sequence of using epidemiology to generate hypotheses for testing in clinical trials, the current research points to anthocyanins and flavan-3-ols as having a selective role in reducing development of T2DM. Subsequent sections provide a summary of the clinical evidence, and specific preclinical research for these two flavonoid classes, and explore the evidence of flavonols and isoflavones in T2DM risk reduction.

### 5.1. Dietary Anthocyanins and Reducing Development of T2DM 

#### 5.1.1. General Background

Anthocyanins are water soluble pigment compounds responsible for the blue, red and purple colors in most fruits and vegetables. They are commonly found in red to purplish blue-colored fruits, vegetables, grains and beans and some roots. The human diet includes six main anthocyanidins (aglycone): cyanidin, delphinidin, malvidin, pelargonidin, peonidin, and petunidin. Glycosylation (i.e., anthocyanin) adds to their chemical complexity and influences their stability, absorption patterns and metabolic fate [57]. Anthocyanin content and composition varies greatly in plant foods [26] with berries being the most anthocyanin dense in the diet. Color is a general guide to which anthocyanins are present, which may be important for dietary guidance as research identifies specific effects of certain anthocyanins and/or their metabolites in T2DM risk reduction. Average daily intake of anthocyanin is ~10.3 mg/day in the USA [24]. A higher intake of anthocyanins has been reported in Italy (44.1–64.9 mg/d) [38] and Finland (47 mg/d) [58], which may be explained by higher berry consumption [59] compared with berry intake in the USA [60]. 

#### 5.1.2. Preclinical Research

Proof of concept and mechanistic studies have been conducted in varied cell culture and animal models to understand the role of anthocyanins in T2DM. Figure 3 illustrates many of the mechanisms ascribed to anthocyanins and their metabolites. This area has been intensively reviewed previously [50,61,62,63]; therefore, the current paper is focused on work since 2010.

Extending earlier research to include the effects of metabolites, Scazzocchio et al. reported cyanidin-3-O-β-glucoside and its metabolite protocatechuic acid upregulated PPAR-γ activity, glucose transporter type 4 (GLUT4) translocation, and enhanced glucose uptake in human omental adipocytes, as well as murine 3T3-L1 differentiated adipocytes [54]. Using human HepG2 cells, a model system to study liver metabolism, including xenobiotic metabolism, an anthocyanin-rich mulberry extract was shown to alleviate insulin resistance and increase glucose uptake and glycogen synthesis via activation of PI3K/AKT pathways [64]. Improvements in metabolic parameters were verified in db/db mice supplemented with the mulberry extract [64]. Anthocyanins may also reduce absorption of glucose through glucose transporter 2 (GLUT2) and the sodium-glucose linked transporter 1 (SGLT1) as suggested by some studies [65,66,67]. They have also been reported to stimulate insulin secretion through activation of l-type voltage-dependent Ca^2+^ channels [68] and by the activation of the free fatty acid receptor-1 [69].

Feeding studies in rodents have provided further insight into the role of anthocyanins in T2DM. Kurimoto et al. reported that dietary black soybean seed coat extract rich in anthocyanins improved glycemia and insulin sensitivity in a T2DM mouse model, and effects were related to the activation of AMPK [70]. The activation of AMPK in the skeletal muscle and liver was accompanied by upregulation of GLUT4 in skeletal muscle and downregulation of gluconeogenesis in the liver. Xing et al. [71] also observed increased insulin sensitivity consistent with increased AMPK phosphorylation in white adipose tissue of obese mice supplemented with 5% freeze dried raspberry powder for 12 weeks. In another study, 80 days supplementation of blackberry extract in standard rat diet compared to standard diet alone increased insulin sensitivity, and decreased concentrations of glucose and insulin; however, the changes were greater in female than in male rats [72]. Tani et al. performed an intraperitoneal glucose tolerance test in six-week-old male Sprague–Dawley rats after oral administration of blackcurrant extract vs. control diet (no blackcurrant) and reported increased glucagon like peptide-1 (GLP-1) and insulin secretion [73]. Including purified blueberry anthocyanins in drinking water of mice fed a high fat diet for 72 days significantly lowered fasting glucose concentrations and corrected β-cell function compared to control mice fed a high fat diet only [68]. In contrast, including blueberry juice did not show the same effect as the purified anthocyanins in the same study setting, which could have been a result of differences in sugar intake in juice vs. anthocyanins supplemented water, lower dose of anthocyanins consumed in drinking water and or other components in blueberry juice that may interfere with anthocyanin mechanism of action [74]. 

#### 5.1.3. Clinical Research

Available literature on the effect of dietary anthocyanins to reduce risk of diabetes in human clinical trials is growing. Supplementation with a juice made from dried strawberry and cranberry polyphenol extracts for six weeks resulted in significantly improved insulin sensitivity measured by hyperinsulinaemic-euglycemic clamp in subjects with insulin resistance [75]. This is the second trial supplementing with an anthocyanin rich food [76] or extract [75] where improvements in insulin sensitivity are reported using clamp methodology, but significant improvements on fasting glucose and insulin concentrations were not observed. These results may explain, at least in part, why other reports supplementing daily for 6–8 weeks with blueberry, bilberry, strawberry or pomegranate did not find improvements on fasting indices [77,78,79,80], but improvements were reported on postprandial indices of glycemic control when strawberry, blackberry, bilberry, blackcurrant or pomegranate, although not blueberries, were consumed with bread [81,82], with a meal [83,84,85] or a sugar drink [86,87,88,89]. Postprandial trials from our lab with strawberries suggest simultaneous intake with a meal or within 2 h before a meal may be required to elicit an effect on glucose and/or insulin metabolism, providing support for a role of early phase strawberry/berry metabolites in peripheral glucose regulation [83,90,91]. Likewise, the effects of dietary anthocyanins are subject to the amount of anthocyanins consumed relative to the population phenotype. A modest post-meal reduction in insulin concentrations was reported with 10 g (~ 1 cup fresh weight equivalent) of freeze-dried strawberry powder compared to control (0 g) in overweight hyperlipidemic individuals [83]. However, in a dose response study in obese individuals with insulin resistance, a beverage containing 40 g of freeze-dried strawberry powder significantly reduced the post-meal demand for insulin compared to a control beverage devoid of polyphenols, but matched for fiber [92]. Furthermore, statistical evaluation of the dose-dependent strawberry metabolite profiles relative to clinical outcomes indicated an inverse relationship between the primary anthocyanin metabolites of strawberry and insulin responses and glucose clearance [92]. In individuals with pre-diabetes and insulin resistance, intake of 250 g of frozen red raspberry (~2 cups) in a breakfast meal significantly reduced peak and postprandial (2 h) glucose concentrations compared to control (0 g of red raspberry), whereas inclusion of either 125 g or 250 g raspberry in the breakfast reduced postprandial insulin concentrations compared to control [93]. The aforementioned findings are corroborated by two recent meta-analyses of randomized controlled trials testing anthocyanin intake/anthocyanin rich foods on cardio-metabolic risk factors. The meta analyses indicated that the effects of anthocyanins on glucose homeostasis measures, including fasting glucose and hemoglobin A1c (HbA1c, an indicator of postprandial and fasting glucose control), and insulin sensitivity/ resistance (homeostasis model assessment index-insulin resistance, HOMA-IR) depends on the dose of anthocyanins, body mass index (BMI)/population phenotype, and anthocyanin source [94,95], specifically highlighting the beneficial effects of berries as a source of anthocyanins.

Intervention studies examining the effects of anthocyanin intake in people with T2DM are limited but demonstrate benefits. In one study, 58 patients with T2DM were given 160 mg of anthocyanins twice daily or placebo (*n* = 29/group) for 24 weeks in a randomized, placebo-controlled, double-blind trial [96]. Supplementation with anthocyanins decreased fasting glucose and insulin resistance (measured by HOMA-IR) compared to placebo [96]. Moazen et al. reported that daily intake of 50 g strawberry powder for six weeks significantly decreased HbA1c in newly diagnosed T2DM patients (*n* = 19) compared to matched subjects in control group (*n* = 17) [97].

Overall, there appears to be complementary data from the collective epidemiological and human clinical trial investigations suggesting that dietary anthocyanins have a strong potential to modulate the risk of T2DM in humans. These findings are further supported by a number of preclinical studies in animals and cell culture models indicating that anthocyanins can work through various cellular signaling pathways, many of which are redox sensitive, to achieve glucose homeostasis.

### 5.2. Dietary Flavan-3-ols and Reducing Development of T2DM 

#### 5.2.1. General Background

The structure of flavan-3-ols is similar to other flavonoids, and like other flavonoids have subclasses that include flavan-3-ol monomers (catechin, epicatechin, epigallocatechin, epicatechin 3-gallate, epigallocatechin 3-gallate, gallocatechin, and catechin 3-gallate), and proanthocyanidins, which range in complexity, but principally represent polymerization of monomeric flavan-3-ols (dimers, trimers, 4–6 mers, 7–10 monomers, polymers) and theaflavins [98,99]. Flavan-3-ols are found in a number of plant foods, including broad beans (average concentration of 154.5 mg total flavan-3-ols/100 g fresh weight); some fruits (ranging from 10 mg to 50 mg/100 g fresh weight) such as plum, apple, custard apple, strawberry-tree fruit, berry fruits such as blueberry and cranberry, cherry, grapes, red wines, cocoa/chocolate (185 mg/100 g of cocoa powder); and green tea and black tea (43.8 and 26.8 mg/100 ml of infusion, respectively) [100,101]. Very high concentrations of flavan-3-ols (polymers) can be found in nuts (hazelnuts—500 mg/100 g; pecans—494 mg/100 g; pistachios—237 mg/100 g; almonds—184 mg/100 g) [102]. Epidemiological research suggests intake of flavan-3-ols ranges from 22–28 mg/d in the USA [32,34]. 

#### 5.2.2. Preclinical Research

Similar to anthocyanins, several epidemiological studies suggest a role of flavon-3-ols in reducing T2DM risk. The preclinical research provides insight into potential mechanisms of action, which includes free radical scavenging activity, mediation of inflammatory responses and enzymes involved in glucose metabolism. Some of these activities are noted in Figure 3. Briefly, cell culture studies and animal studies have shown flavan-3-ols, such as epigallocatechin-3-gallate (EGCG) and extracts from cacao containing multiple flavan-3-ol subtypes, influence oxidative stress, inflammation and glycemic control pathways [103,104,105]. For example, EGCG inhibited Cd^2+^ induced apoptosis of human liver cells acting as a reactive oxygen species scavenger [103], and attenuated inflammation-induced insulin resistance in 3T3-L1 adipocytes [104]. Also with EGCG, insulin sensitivity was increased, c-Jun N-terminal kinases (JNK) phosphorylation (p-JNK) was suppressed and GLUT4 expression was increased in the adipose tissue of EGCG supplemented obese KK-ay mice and high-fat diet-induced obese rats [104]. The effects in these animal models coincided with reduced glucose concentrations and improved glucose tolerance [104]. Supplementing cacao extract in the diet of high fat fed mice promoted GLUT4 translocation and increased activation of AMPK in the plasma membrane of brown adipose and skeletal muscle, consistent with reduced hyperglycemia, glucose intolerance and fat accumulation [52]. Thus, in vitro and in vivo models suggest that both monomeric and mixtures of flavan-3-ols subtypes influence mechanisms important in glucose control. 

#### 5.2.3. Human Studies

Extending the preclinical research, there is considerable evidence supporting the effects of dietary flavan-3-ols on metabolic indices of glucose and/or insulin metabolism. These data are found in recent meta-analyses of randomized clinical trials examining the effects of cocoa product intake on cardio-metabolic risk factors in varied population groups [106,107,108]. Reported findings suggest modest but significant improvements on insulin-related outcomes, including decreased fasting insulin and improved insulin sensitivity assessed by HOMA-IR. Furthermore, intake of > 200 mg per day flavan-3-ols achieved benefits for insulin endpoints, whereas doses between 200–600 mg flavan-3-ols per day were associated with reduced glucose concentrations [108]. In postmenopausal women with T2DM, 1-year dietary intervention with flavonoid-enriched chocolate (850 mg flavan-3-ols/d) combined with isoflavones (100 mg/d) resulted in significant improvements in insulin sensitivity and reduced insulin concentrations, demonstrating additional benefits of a flavan-3-ols/isoflavone mixture to standard drug therapy on various cardio-metabolic risk endpoints [109]. Another study demonstrated insulin-specific benefits in adults with essential hypertension and impaired glucose tolerance after 15-day supplementation with 100 g flavan-3-ol-rich dark chocolate [110]. Using grape seed extract (GSE) as a source of flavan-3-ols, individuals with pre-hypertension who drank beverages containing 300 mg GSE flavan-3-ols vs. control (0 mg) daily for 6 weeks showed trends in decreased fasting insulin and increased insulin sensitivity; a benefit that regressed after discontinuation of the beverages for four weeks [111]. Tea, as a source of EGCG, has also been investigated for effects on T2DM endpoints revealing positive effects, including decreased HbA1c values compared to baseline measures in adults with impaired glucose tolerance [112]. Improved HbA1c and insulin sensitivity in individuals with T2DM have been reported [113,114]. In contrast, other clinical studies demonstrated that EGCG had no effect on insulin sensitivity [115] or glucose tolerance in obese adults with metabolic syndrome [116]. Overall, including foods rich in flavan-3-ols in the diet as a strategy to reduce risk of and manage T2DM seems advantageous. Similar to results from the dietary anthocyanin literature, the collective findings warrant follow-up testing for longer duration and dose finding trials to develop clear dietary recommendations for individuals with diabetes concerns. 

### 5.3. Other Possible Flavonoids That Reduce Development of T2DM 

Flavonols and isoflavones have surfaced as having possible anti-diabetic actions. In a recent meta-analysis, data analyzed from seven studies (nine cohorts) identified flavonols (as a class but not individual flavonols i.e., querecetin, kaempferol) correlated with lower incidence of T2DM, whereas analyses on isoflavones using six studies/eight cohorts indicated both the class (isoflavones) and individual subclass compounds (i.e., genistein and daidzein) were inversely associated with diabetes risk [31]. Preclinical data, including animal and in vitro studies, have demonstrated effects of flavonols, mainly quercetin, on a number of diabetes-related processes [55,117,118,119] and mechanisms related to flavonoid action as shown in Figure 3. However, the human clinical data are limited and have largely not substantiated the preclinical work to date [117]. 

In vitro studies with genistein [56] have shown effects on pancreatic β-cell proliferation, glucose-stimulated insulin secretion and protection against apoptosis, and these effects are independent of genistein’s activity as an estrogen receptor agonist, antioxidant, or tyrosine kinase inhibitor [56]. However, evidence of the effects of purified genistein intervention in humans with T2DM is extremely limited, despite studies indicating it’s safe for human consumption [120]. Isoflavone mixtures or soy are often studied instead, although still limited in establishing a clear relationship between isoflavones and T2DM risk reduction. For example, isoflavone supplementation (100 mg) for six months in a placebo controlled study in pre-diabetic or early untreated T2DM women showed no benefit on fasting glucose, 2 h glucose or HbA1c compared to placebo control, or soy protein with isoflavones [121]. Whereas earlier work (2002) in post-menopausal women with a slightly higher dose of isoflavones (132 mg) showed decreased HbA1c, fasting insulin and reduced insulin resistance as measured by HOMA-IR [122]. A meta-analysis of randomized control trials investigating phytoestrogen supplementation and body composition in postmenopausal women suggested that phytoestrogen supplementation is associated with reduced weight in healthy postmenopausal women, but increased body weight in postmenopausal women with pre-existing conditions, such as pre-diabetes [123]. With the association between body weight and T2DM risk, dietary supplementations that increase body weight would be counterproductive. 

## 6. Conclusions

Interest in flavonoid health benefits has rapidly increased over the last decade. Advancements in instrumentation have enhanced the field’s ability to identify and quantify these compounds in foods, linking their intake with disease risk and possible mechanisms of actions in T2DM. The collective epidemiological research suggests that select flavonoids, specifically dietary anthocyanins and flavan-3-ols, have an important role in T2DM risk reduction. Controlled clinical trials testing purified compounds, extracts, and foods rich in anthocyanins and flavan-3-ols have provided additional evidence verifying beneficial bioactivity on endpoints important in T2DM development. Future work pursuing metabolite characterization and kinetic profiling linked with clinical biomarkers of disease risk after dietary flavonoid ingestion will help illuminate the preventative and therapeutic role of these compounds, particularly in understanding responses in the context of population characteristics, dose, and food vs. supplement effects. These data will be critical for devising dietary guidance that is targeted and efficacious for long-term metabolic health. 

Overall, the research indicates that dietary flavonoids do not universally influence diabetes risk. Instead, selective flavonoids, explicitly anthocyanins and flavan-3-ols and foods rich in these compounds, are biologically active on mechanisms underlying risk factors of T2DM. Early intervention in *at risk* individuals with diets focused on increasing anthocyanin and flavan-3-ol intake may be particularly opportunistic in reversing or reducing the disease risk trajectory preventing advancement to pre-diabetes and further to T2DM.
